# Face-to-face more important than digital communication for mental health during the pandemic

**DOI:** 10.1038/s41598-023-34957-4

**Published:** 2023-05-17

**Authors:** S. Stieger, D. Lewetz, D. Willinger

**Affiliations:** Department of Psychology and Psychodynamics, Karl Landsteiner University of Health Sciences, Krems an der Donau, Austria

**Keywords:** Psychology, Human behaviour

## Abstract

During the lockdowns associated with the COVID-19 pandemic, many people tried to compensate for limited face-to-face interaction by increasing digital communication. Results of a four-week experience sampling study in the German-speaking countries (*N* = 411 participants; *k* = 9791 daily questionnaires) suggest, however, that digital communication was far less relevant for lockdown mental health than face-to-face communication. Digital text-based communication (e.g., e-mail, WhatsApp, SMS) nevertheless was meaningfully associated with mental health, and both face-to-face and digital text communication were more predictive of mental health than either physical or outdoor activity. Our results underscore the importance of face-to-face communication for mental health. Our results also suggest that videoconferencing was only negligibly associated with mental health, despite providing more visual and audible cues than digital text communication.

## Introduction

In 2020, half of the world’s population was ordered or asked by their governments to stay at home in order to curtail the spread of COVID-19^1^. Lockdowns and other restrictions on face-to-face interaction were more or less successful in containing the COVID-19 virus^2^, but they also had severe negative effects on different indicators of mental health such as depression, anger, fear and post-traumatic stress (for reviews, see^3,4^). To ameliorate the mental strain caused by the imposed restrictions, experts and governments encouraged people to maintain social contact while also maintaining physical distance^5^. Indeed, many people increased the use of digital text-based communication, videoconferencing, and telephone calls to keep in touch with friends and family during the lockdowns^6–8^. But it remains unclear if these diverse digital communication possibilities can indeed be a substitute for a face-to-face communication when it comes to people’s mental health and if yes, how could this be explained.

Media Richness Theory (MRT;^9^) states that the degree of nonverbal cues (especially the immediacy) is the most important prerequisite whether or not people build relationships through technology. For example, in a communication situation with few to virtually no social cues (e.g., mere text-based communication), interaction partners will experience their communication partner as less empathic or friendly^9^. This means that the social presence of the communication partner is vital for building interpersonal relationships. This is also the reason why people sought to compensate for the absence of social presence in text-based communication by resorting to visual aids, such as emojis, memes, and photos to enrich the message with supplementary social cues (e.g., emotions;^10^). Due to the technological progress, modern technology-mediated communication means such as video-conferencing offer a very rich environment of social cues compared to face-to-face communication. With the advent of high-speed internet technologies, such as fiber-optic connections and 5G mobile networks, we are now endowed with the ability to engage in virtual conversations with vivid imagery and accurate tone, nearly approximating the quality of in-person communication, and with very low time lags. Although face-to-face communication is currently considered ‘the gold standard’^11^, the question arises if current forms of communication such as video-conferencing can already match this benchmark in terms of promoting people's mental and emotional well being.

In a recent large cross-sectional study by Hall et al.^12^ conducted during the shelter-in-place orders, it was found that face-to-face contact outside of home was an important predictor of well-being (e.g., lower loneliness) although also contradicting results based on the MRT were found (voice calls positive for well-being, video chats negative). Another study using an experience sampling methodology (ESM) design by Kushlev et al.^13^ tried to analyze two hypotheses. First, the complementary hypothesis which stated that digital communication complements face-to-face communication and therefore heightens well-being, and second the interference hypothesis which assumes that face-to-face communication impoverishes by additionally using digital communication (i.e., social displacement theory). Indeed, they found that when both forms of communication were used, participants felt less connected and worse compared to when solely using face-to-face communication. This was also found by Verduyn et al.^14^ again in an ESM design, although the study generally focused on smartphone-based communication vs. face-to-face communication. Face-to-face communication was positively associated with positive affect, and negatively associated with negative affect; smartphone-based communication—and social media communication in particular—vice versa.

Another recent large cross-cultural study by Newson et al.^15^ followed a similar rationale by asking if computer-mediated communication can make up the reduced face-to-face communication during lockdown situations. Again, they found that face-to-face contact was positively associated with well-being whereas communication using messaging apps (e.g., email, social media) was negative. Interestingly, phone calls and video chats had no significant association with well-being. Petrova and Schulz^16^ again used the COVID-19 pandemic situation to analyze the impact of different forms of communication (from most life-like communication, i.e., face-to-face to least life-like communication, i.e., text-based) onto well-being by using an ESM design. They found that the more life-like the communication was, the less lonely, less sad, more affectionate, more supported, and more happy people felt when compared to less life-like situation. All the more, this association was so strong for loneliness and happiness that already between face-to-face communication and the closest life-like situation, i.e., video-calls, a significant difference was prevalent. To sum up, although findings are mixed, they rather support the notion that technology-mediated communication is not capable to be a substitute for a face-to-face communication.

In the present study, we used experience sampling methodology^17–19^ to examine the extent to which face-to-face and digital communication were related to mental health during the lockdowns associated with the COVID-19 pandemic. We use the term Experience Sampling Method as an umbrella term to reflect the many different facets this assessment method has. Other terms are Ecological Momentary Assessment (EMA), Ambulatory Assessment (AA), and diary method to name just a few (for a discussion about this, see^20^). We made use of the lockdowns in the German-speaking countries, where government measures required people to stay at home and physically distance themselves from people outside of their household. The lockdown situation constitutes a natural field experiment during which face-to-face and digital communication as well as mental health are likely to fluctuate. We asked a diverse and well-powered sample of people experiencing any form of lockdown restriction to report their mental health; time spent on face-to-face communication; and time spent on digital text-based communication (e.g., e-mail, chats, WhatsApp, SMS), videoconferencing (e.g., Skype, Zoom), and telephone calls at the end of each day for four weeks (*N* = 411 participants, *k* = 9791 completed daily questionnaires). As two other activities known to be related to mental health^21,22^ and promoted as strategies for coping with the lockdown, participants also reported how long they spent on physical and outdoor activity each day for use as control variables and frames of reference regarding potential effect sizes. This is also relevant, because the time used for sports and outdoor activity usually reduces the time for other communication possibilities (e.g., face-to-face communication) especially during the times of lockdown, because many restrictions had the focus not to meet other people, e.g., team sport were not allowed.

## Results

### Validity, reliability, and dropout

Our analyses indicated high data quality. Demographic data assessed at the beginning of the study and again after four weeks were highly consistent (gender: Cohen κ = 0.939; age: intraclass correlation, ICC > 0.99, *r* = 0.99, *p* < 0.001; relationship status: Cramer ν = 0.913; nationality: Cramer ν = 0.975). Our measure of mental health—which was adapted for daily assessments via smartphone—was moderately correlated with somatic symptoms assessed at the end of the study (*r* = − 0.40 across all data points). This is in line with previous research on the original measure of mental health which refers to the last four weeks (*r* = − 0.27;^23^). Furthermore, our daily measure of mental health was substantially correlated with a trait-measure of mental health assessed at the end of the study (*r* = 0.79). Both results speak for the criterion and construct validity of the daily mental health measure used in the current study. Based on Generalizability Theory Analysis^24,25^, we found that the mental health measure had satisfactory within-person reliability (*R*_C_ = 0.67–0.76) and excellent between-person reliability (*R*_kR_ = 0.98–0.99), indicating that we reliably assessed both day-to-day variation as well as inter-individual differences in mental health.

A total of *N* = 864 people started the study but dropped out early or took part only sporadically, which is rather common for app-based assessments^26^. We defined completion of fewer than seven questionnaires as drop out/low compliance. Dropout can be a problematic if the people who dropout systematically substantially differ from those who fully participate^27^. There was, however, no evidence that dropout was related to gender (χ^2^ = 0.677, *p* = 0.713), age (*t* = − 0.852, *df* = 845, *p* = 0.394), relationship status (χ^2^ = 3.976, *p* = 0.553) or nationality (χ^2^ = 6.452, *p* = 0.092). Only small differences have been found for the study’s main variables with varying directions (significant: MHC-SF β = − 0.13, videoconferencing β = 0.10; text-based communication β = 0.06; face-to-face communication β = − 0.07; non-significant: telephone communication β = 0.02; physical activity β = − 0.02; outdoor activity β = 0.02).

### Descriptive analyses

Overall, mental health was intermediate across participants and days (*M* = 8.47 out of a possible 14). Participants spent *M* = 296.2 min per day (~ 5 h) on face-to-face communication (*SD* = 293.3), *M* = 57.7 min per day on digital text communication (*SD* = 147.6), *M* = 44.9 min per day videoconferencing (*SD* = 153.6), and *M* = 32.8 min per day on telephone communication (*SD* = 133.6). Mental health fluctuated a lot over the four-week period (*SD* = 4.21), but mental health was uncorrelated with the day of assessment (random-effects multi-level linear regression analysis: *B* < 0.01, *p* = 0.13). Just over one quarter (28%) of the variance in mental health was within-person (i.e., due to day-to-day fluctuations), and 72% of the variance was between-person (i.e., due to stable differences between participants). Time spent on face-to-face and digital communication likewise fluctuated across the four-week period, probably due to the changing lockdown situations (face-to-face communication: ICC = 0.53; digital text: ICC = 0.73; videoconferencing: ICC = 0.68; telephone: ICC = 0.84).

### Research question: associations with mental health

Table 1 displays the results of a multi-level linear regression analysis with daily assessments (level 1) nested in participants (level 2), mental health as the dependent variable, and the log-transformed communication variables as the predictors. As seen in Table 1, our model was able to explain substantial parts of the variance in mental health (*R*^2^_conditional_ = 76%). According to the level 2 results, people who generally spent more time on face-to-face and digital text communication during the lockdown had better mental health than people who generally spent less time on face-to-face and digital text communication (small to medium effect sizes as indicated by the standardized β coefficients) and vice-versa. According to the level 1 results, mental health was also better on days when people spent more time on face-to-face communication than usual (small to medium effect size). To a far lesser extent, mental health was also better on days when people spent more time on digital text and videoconferencing than usual (negligible effect sizes). Interestingly, there was no evidence that telephone communication was related to either between- or within-person variation in mental health. Supplemental analyses revealed only weak evidence that the relationship between the communication variables and mental health may to some extent depend on whether people communicated with family, friends, or others (Table S1). Furthermore, we analyzed if the pattern of results remains when only analyzing participants who have stated being single and living alone. For those participants, the lockdown situation was probably more impactful, because a face-to-face communication within their direct living environment was not possible. As can be seen from Table S3, the main conclusions basically remained except for two differences. First of all, the communication using a telephone was also a significant positive predictor for good mental health, and second also participant’s age was substantially associated with mental health. The younger participants were, the lower was their mental health and this association was even quadratic, i.e., younger participants were probably substantially more affected by the lockdown situation than older ones. These results have to be interpreted with caution, because the subsample size was substantially lower (*k* = 3793) compared to the overall sample.

**Table 1:** Results of the multi-level regression analysis of face-to-face and digitally-mediated communication as predictors of mental health.

	Fixed						Random	
	Coeff.	*B*	*CI B*	β	*SE B*	*t*	Coeff.	*SD*
Intercept	β_00_	3.58	1.91 to 5.25		0.85	4.20***	*r*_0*i*_	3.22
Within-person (all .cwc)								
Face-to-face^a^	β_10_	0.31	0.26 to 0.37	0.21	0.03	11.93***	*r*_1*i*_	0.32
Videoconferencing^a^	β_20_	0.05	0.03 to 0.08	0.04	0.01	4.06***		
Digital text^a^	β_30_	0.06	0.01 to 0.10	0.03	0.03	2.17*	*r*_3*i*_	0.24
Telephone^a^	β_40_	0.03	− 0.00 to 0.06	0.02	0.01	1.92		
Sport activity^a^	β_50_	0.09	0.06 to 0.11	0.07	0.01	6.09***		
Outdoor activity^a^	β_60_	0.13	0.10 to 0.16	0.09	0.02	8.20***		
Between-person (all .pm, except gender and age)								
Face-to-face^a^	β_07_	0.71	0.45 to 0.98	0.27	0.13	5.29***		
Videoconferencing^a^	β_01_	0.24	− 0.03 to 0.50	0.09	0.14	1.73		
Digital text^a^	β_04_	0.51	0.18 to 0.85	0.16	0.17	3.00**		
Telephone^a^	β_010_	0.08	− 0.24 to 0.40	0.03	0.16	0.52		
Sport activity^a^	β_013_	0.37	0.07 to 0.67	0.14	0.15	2.39*		
Outdoor activity^a^	β_014_	− 0.24	− 0.62 to 0.13	− 0.08	0.19	− 1.27		
Gender (female)^b^	β_015_	− 0.88	− 1.74 to − 0.02	− 0.09	0.44	− 2.00*		
Age.cgm	β_016_	− 0.03	− 0.07 to 0.02	− 0.09	0.02	− 1.18		
(Age.cgm)^2^	β_017_	< 0.01	− 0.00 to 0.00	0.11	< 0.01	1.57		

*Note*. *R*^2^_conditional_ = 76%, *R*^2^_marginal_ = 13%; AIC = 42,435, BIC = 42,600, Ω^2^ = 79%.

cwc, centered within cluster; pm, personal mean; cgm, centered around grand mean.

*p < 0.05, **p < 0.01, ***p < 0.001.

^a^Log transformed.

^b^Reference group for gender was male.

As an alternative method for assessing the relative importance of face-to-face and digital communication for lockdown mental health, we additionally calculated a mixed-effects random forest (MERF) model^28,29^. In general, random forest (RF) models use recursive partitioning^30^ to assess the importance of each predictor on a particular outcome by analysing all possible relationships between the predictors and the outcome. This is done by drawing random subsets of predictors and participants, examining the predictive power of each predictor within the respective subset, and repeating the procedure over hundreds of bootstrap samples. MERF is a special adaptation of RF models for longitudinal data^29^. MERF and RF models have the advantage that they are unaffected by either multicollinearity or non-linearity, and they can flexibly account for complex interactions between predictors. Since MERF and RF models are based on a non-parametric procedure, no log-transformation of the communication variables was required, which also makes it easier to directly compare their predictive power.

**Figure 1:**
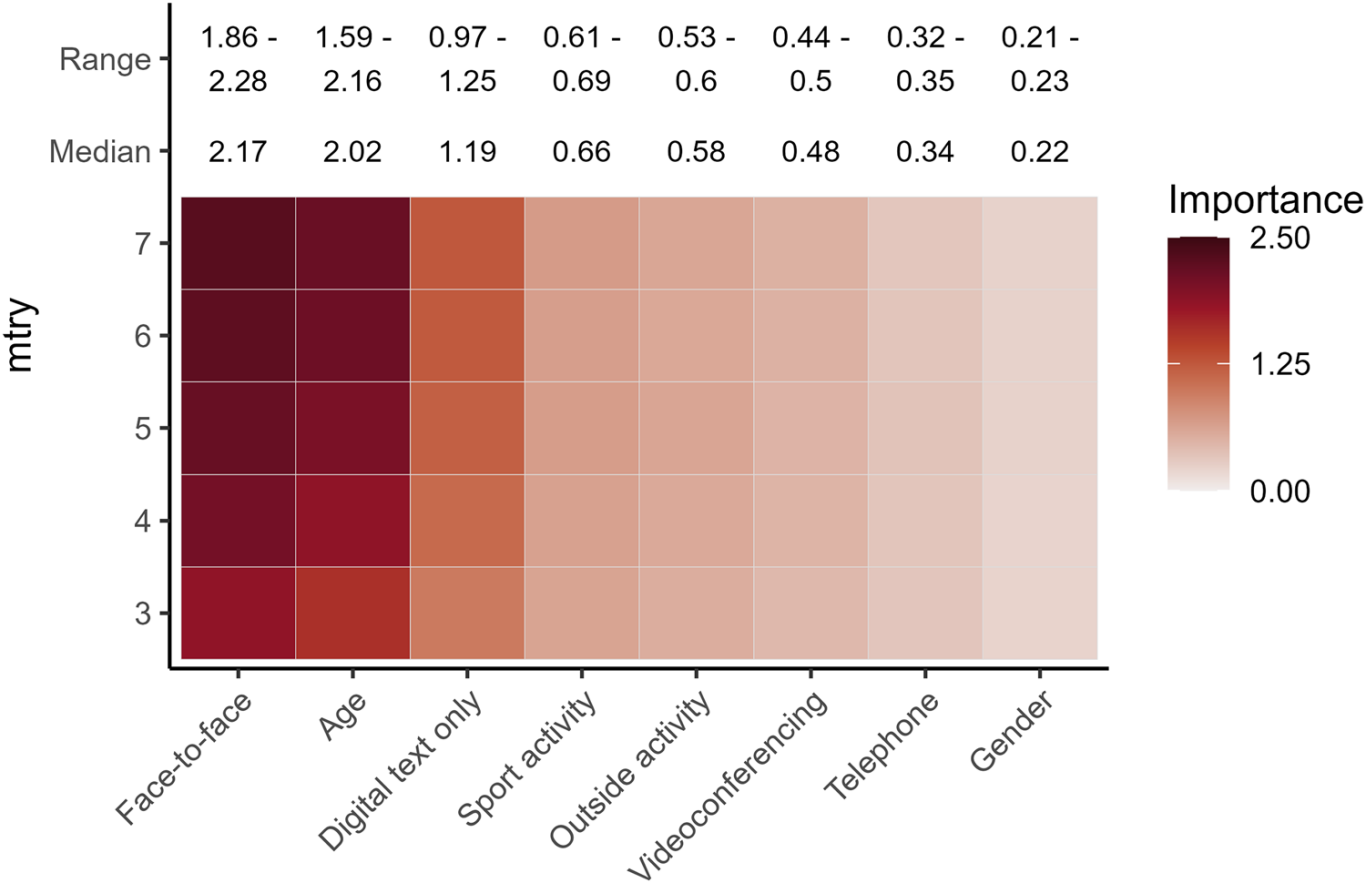
The variables in order of importance from the mixed effects random forest models with different *mtry* parameters.

According to the MERF analysis, the most important predictor of lockdown mental health was face-to-face communication, followed by age and digital text communication. Notably, face-to-face and digital text communication were found to be more predictive of mental health than physical and outdoor activity (Fig. 1) as established positive predictors of mental health^21,22^. Videoconferencing, telephone communication, and gender were much less predictive of mental health. The outcome of MERF models may depend on the number of predictors (randomly selected from all predictors in the model) included in each set (i.e., the *mtry* parameter). The rank order of the variables with regard to their predictive power remained the same regardless of whether *mtry* was set from 3 to 7 (see the similarity of the lines in Fig. 3). The MERF model explained between 22.7 and 23.7% of the overall variance in mental health, depending on *mtry*.

## Discussion

Our results are clear: face-to-face communication was much more important for lockdown mental health than digital communication. Similarly, a longitudinal study from the advent of the Internet in the 1990s found that more Internet use led to less face-to-face contact and increases in depression and loneliness^31^—it seems that this overall picture has not much changed until today. The multitude of digital communication devices and services available in the Western world still appear to be unequal substitutes for face-to-face interaction still being ‘the gold standard’^11^. That said, our results also suggest that digital text communication was meaningfully predictive of mental health, albeit to a lesser extent than face-to-face communication. Interestingly, both face-to-face communication and digital text communication were stronger predictors of lockdown mental health than either physical and outdoor activity—two established positive predictors of mental health^21,22^.

Why is face-to-face communication so much more important for mental health than digital communication? Researchers have long noted that far less information about the social context is available in the digital than in the face-to-face setting, such as the cues about the personality and social status of the communication partners (as displayed through e.g., clothing and behavior), or social norms (e.g., who sits where in a conference room). The depersonalization and deindividualization of communication partners in the digital setting may explain why digital communication is less relevant for mental health than face-to-face communication (depersonalization theory;^32^). Social engagement and attachment theory^33^ suggests that cues such as body language, voice pitch, mimic, eye gaze, and head position allow both the expression and reception of social cues, which in turn reduce the perceived psychological distance between communication partners. Digital communication may not activate the largely subconscious, neurophysiological tools which have evolved in order to help humans determine who is friend, and who is foe (cf.^34^).

Against the backdrop of depersonalization theory as well as social engagement and attachment theory, it is curious that digital text communication was much more predictive of lockdown mental health than videoconferencing, even though videoconferencing allows communication partners to experience many more visual and audible cues than digital text communication. Recent research and anecdotal reports show that videoconferencing can cause adverse effects such as mental tiredness (‘Zoom fatigue’;^35^); anxiety due to a focus on appearance, prolonged eye contact, larger faces due to screen size, and the perceived dominance of a communication partner due to low camera position; and cognitive burden due to the slight technological asynchrony of video calls (for a review, see^36^). Furthermore, it could be that in our sample videoconferencing was predominantly used in work situation and less private situations because many companies sent their employees into home-office to work from there. More detailed research on the mental health costs and benefits of videoconferencing is urgently needed, particularly because videoconferencing is increasingly discussed as an effective means for delivering psychotherapy (e.g.,^37^) and telehealth (e.g.,^38^). A further important avenue for future research may be to explore whether using virtual reality glasses to interact with an avatar of a communication partner would have a similar effect on mental health as face-to-face communication, as the interaction would be experienced visually as well as physically.

While our study yields important insights, it is not exempt from limitations. First, our initial power calculation was based on 28 retests but in fact the mean number of daily assessments for those taking part for 28 days was *M* = 19 (*SD* = 7.6). Nevertheless, recalculating the power analysis with 19 retests only slightly heightened the needed sample size to *N* = 263 and was still lower than the actual recruited sample size. Second, we could not control for changes in lockdown situations. Lifting restrictions (e.g., no curfews anymore) probably was positive for mental health independent from communication patterns and physical activity. Changes in lockdown situations was impossible to plan for, as we naturally could not know when new restrictions would be introduced, or existing ones lifted. Furthermore, as already mentioned above, investigating differences between private and work-based communication would also be an area worth pursuing in future studies.

## Conclusion

In conclusion, despite living in a highly technological world, particularly in industrialized western nations, the numerous technological devices and services available cannot replace the mental health and well-being benefits of in-person communication. The future will show whether further technological advances, such as 5G or virtual reality, can elevate our online social communication to a level comparable to a real-life face-to-face interaction.

## Methods

### Power calculation

We expected small effect sizes of *r* = 0.1 between the communication variables and mental health. A rough power estimation based on the recommendations from Twisk (^39^, p. 123ff) indicated that a sample of around *n* = 254 was needed (assumed intraclass correlation, ICC = 0.3, α = 5%, power = 80%, two-sided, number of assessments: ~ 28 over a period of 4 weeks). To account for non-response and dropout (~ 10–15%), we aimed to recruit at least 300 participants.

### Participants

The project was conducted in accordance with the ethical standards of the institutional and/or national research committee and with the 1964 Helsinki Declaration and its later amendments or comparable ethical standards. The research received ethical approval from the Commission for Scientific Integrity and Ethics of the Karl Landsteiner University of Health Sciences, Austria (IRB approval number: 1086/2020). Informed consent was obtained from all participants after the nature and possible consequences of the study were explained.

Participants were recruited through diverse channels including advertisements in printed newspapers, postings on Facebook and other social media channels, student pools at several universities, and so forth. There were no exclusion criteria except that participants had to be 18 years of age or older. Participants did not receive any financial compensation. As an incentive, participants could access graphical feedback on a selection of their own data (e.g., mental health over time), as well as graphical feedback on the whole sample of participants (e.g., gender ratio in the whole sample, number of completed questionnaires) (cf.^40,41^).

**Figure 2:**
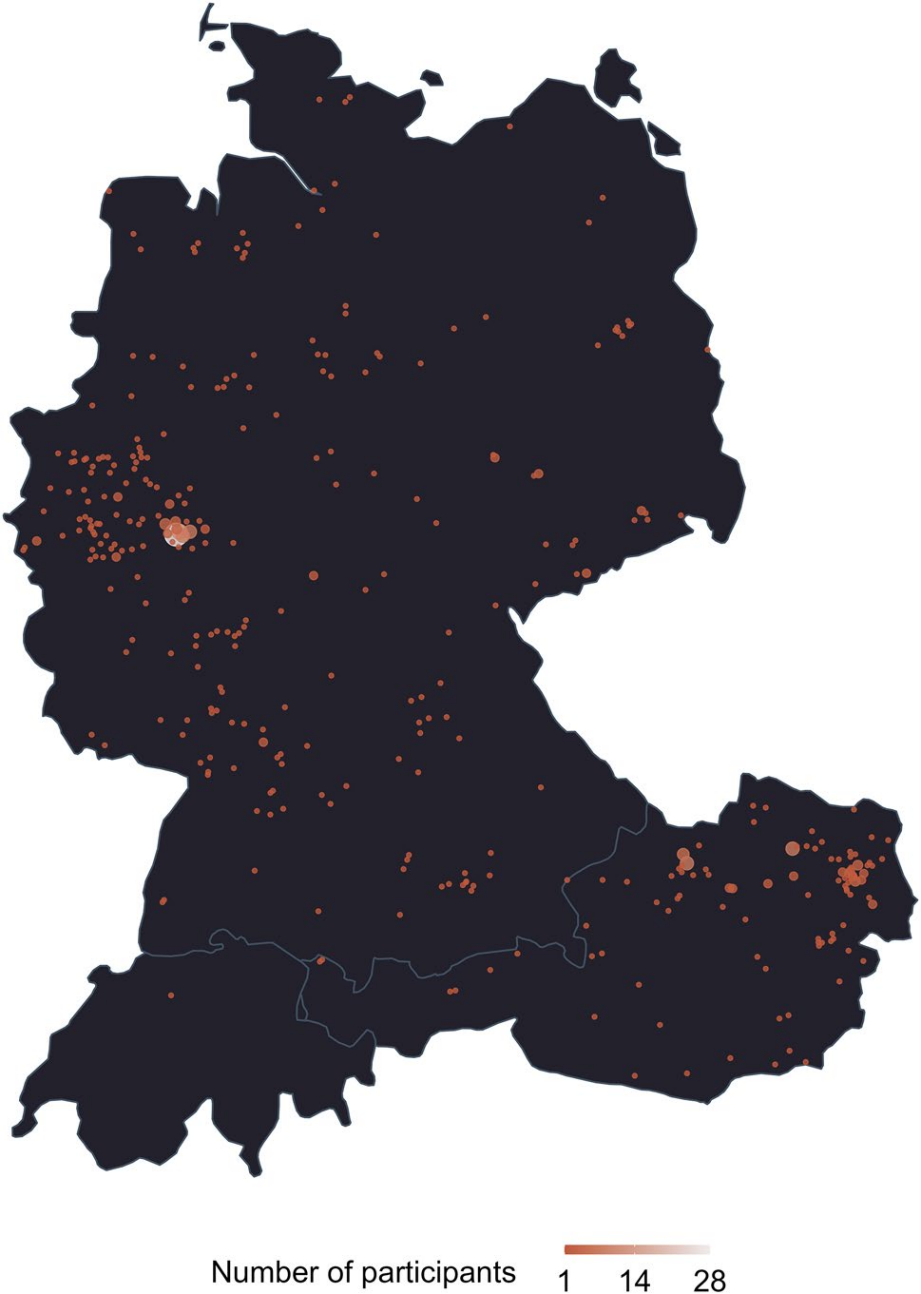
Dispersion of participants across Germany, Austria, and Switzerland.

**Figure 3:**
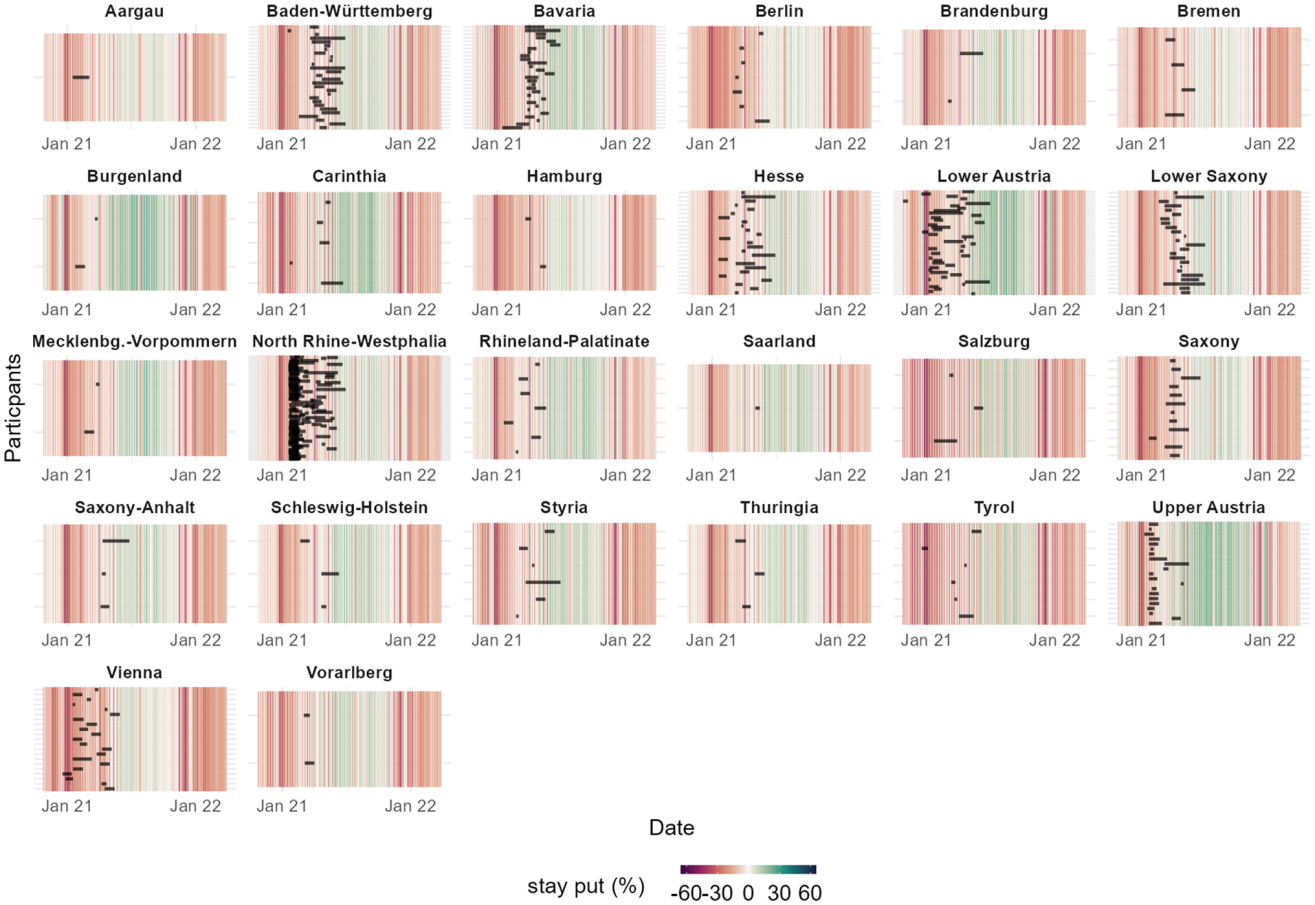
Near real-time mobility data provided by Facebook confirms that mobility was dramatically decreased in the region of all those participating in the study. Each black bar corresponds to the duration of participation of one individual in the respective region. The colors depict the decrease or increase in mobility relative to February 2020.

Participants were dispersed across the German-speaking countries (predominantly Austria and Germany; see Fig. 2) from different states and areas (e.g., urban vs. rural) reflecting a community-based sample. Using independent data from Facebook and participants’ self-reported ZIP codes, we confirmed that mobility in all of the regions where participants had been recruited was severely restricted for the duration of their participation. As can be seen in Fig. 3, individual assessment phases (black bars) mostly overlap with times of restrictions (vertical lines colored red).

A total of *N* = 864 participants installed the app and completed the demographic questionnaire at the start of the study, of which 473 completed the questionnaire after four weeks. Since there is no clear recommendation in the experience sampling methodology (ESM) literature, we defined dropout/low compliance as fewer than seven completed daily questionnaires. The cut-off was based on inspection of the response rate curve, which stabilizes after seven completed questionnaires (see Fig. S1). This might represent rather a rough cut-off, but due to a missing clear evidenced-based recommendation in the literature regarding the number of missing data sets that would qualify as an indicator of poor data quality, we opted for this rather lenient criterion to be err on the side of caution. The three participants who indicated diverse gender were excluded from the sample because the group was far too small for further analyses. After excluding participants with fewer than seven completed daily questionnaires and data cleaning procedures (e.g., deletion of double entries, implausible demographics), *N* = 411 participants remained in the final dataset who, in sum, completed *k* = 9,791 end-of-the day questionnaires.

Participants in the final sample were *M* = 32.1 years old (*SD* = 12.50, range: 18–76 years) and predominantly women (83.0%). Most were from Germany (71.3%), Austria (24.6%) and Switzerland (0.7%); 1.9% stated another country (1.5% missing). Regarding participants’ current relationship status, 34.3% were in a relationship, 39.7% single and living alone, and 19.5% were married or in a partnership (4.1% divorced; 0.7% widowed; 1.7% missing).

### Procedure

The study followed ESM. We used the software *ESMira*^42^ for project administration and data collection. ESMira was developed especially for data collection in ESM studies. It offers a wide repertoire of functions for scientific data collection such as the presentation and acceptance of the informed consent form, data security, data encryption, graphical feedback, anonymous chat function, and guaranteed anonymity through randomly generated subject codes.

People interested in participating were directed to a project-specific website which provided more detailed information about the study. They could subscribe to the study by scanning a project-specific QR code after installing ESMira on their own smartphone. ESMira was available for Android and iOS operating systems.

After indicating informed consent and receiving general information about the procedure, participants completed a short demographic questionnaire (~ 5 min) one minute after joining the study. This was done to familiarize participants with the procedure of receiving acoustic bings (also called signals or notifications) and completing questionnaires directly in the app in response.

Data collection took place in participants’ everyday environment (e.g., work, home, university). For the duration of the study, ESMira used an acoustic bing to prompt participants to complete the end-of-day questionnaire at 8 p.m. No reminders were used but the bing was permanent; that is, it did not turn off after a certain time. Participants had the possibility to adjust the time of the bing to suit their individual needs (e.g., shift workers).

The study was open-ended, but participants were encouraged to participate for at least four weeks. Participants could quit the study at any time by using a “quit study” button. After four weeks, ESMira asked participants to complete a final questionnaire in order to check the consistency and validity of a subset of the data, as well as assess a number of concepts unrelated to the present study (~ 20 min). After completing the final questionnaire, participants were informed that the official part of the study was over, but that they could take part as long as they wanted (e.g., in order to get personalized feedback). A total of 113 participants took part for more than four weeks. We closed the study after 104 days (~ 15 weeks) because few participants were still taking part in the study (study was open from October 2020 to June 2021).

Because the study was open to the public and conducted virtually, we ensured that participants were familiar with the study’s administration in two ways. First, participants could use a built-in chat function in ESMira to anonymously contact the principal investigator in the case of questions or technical problems; 9 participants used this feature to exchange a total of 20 messages with the principal investigator. In reverse, the principal investigator could contact participants (who remained anonymous) through an online administration tool of ESMira. Second, we constructed a Frequently Asked Questions (FAQs) web page based on our previous experience with ESM studies (14 FAQs).

### Demographic questionnaire

Participants indicated their gender (female, male, diverse), age (years), current relationship status (single and living alone, in a relationship, married or in a partnership, divorced, widowed), years of education (including repeated classes), and nationality (Austria, Germany, Switzerland, other). Furthermore, participants provided the ZIP code of their current residence as well as the ZIP code where they spent most of their childhood.

### End-of-day questionnaire

We adapted the Mental Health Continuum Short Form measure (MHC-SF;^43,44^; German version:^45^) to assess mental health on the current day. The MHC-SF consists of 14 items which assess three dimensions of mental health: specifically, emotional, social, and psychological well-being. Because recent research^45^ questioned the three-dimensional structure of the MHC-SF in a large cross-cultural study, we only use the overall score of the MHC-SF. Usually, the scale refers to mental health over the past month (e.g., “During the past month, how often did you feel … happy?”) and uses an answer scale ranging from 1 = *never* to 6 = *every day*). To adapt the scale for daily assessments via smartphone, we changed the wording from “past month” to “course of today” (e.g., “During the course of today, how often did you feel … happy?”) and adapted the answer scale to 0 = *never* or 1 = *at least once*. Responses across items were summed, with higher scores indicating better mental health. In order to give participants meaningful graphical feedback about their mental health over time, our adapted measure used a forced-response design; that is, participants had to answer each item in order to proceed). As described below, we used a measure of somatic symptoms and the trait version of the MHC-SF to demonstrate criterion and construct validity of the adapted scale.

Participants also indicated how much time in hours and minutes they spent on that particular day on the following communication channels: face-to-face communication, videoconferencing (e.g., Skype, WhatsApp call), digital text (e.g., e-mail, chats, WhatsApp, SMS), and telephone (cell phone, smartphone, landline telephony). To make the answering process more convenient, participants tapped on the screen to move the hour and minute pointers of a classical, round clock instead of using the keyboard. Participants separately indicated the time spent on each communication channel with family, friends, and other people. To simplify the analyses, we summed the time spent on each communication channel with family, friends and others. Finally*,* participants indicated how much time in hours and minutes they spent on physical activity and outside activity (i.e., outside of a building or means of transport).

The end-of-day questionnaire additionally included measures of screen time and living environment (e.g., rural, urban) which were not part of the current study.

### Final questionnaire

Participants once again indicated their gender, age, relationship status, years of education, and nationality. In order to demonstrate the criterion validity of the adapted mental health scale, we used the Somatic Symptoms Scale-8 (SSS-8;^46^) to assess participants’ somatic symptoms as a behavioral (versus subjective) measure of mental health. The SSS-8 is a reliable and valid short scale of the PHQ-15 which was developed within DSM-5 field trials^47^. It assesses the severity of symptomatic symptoms during the last seven days such as headaches, tiredness, or sleep disturbances by using a five-point Likert-type scale ranging from 0 = *not at all* to 4 = *very strong*. Reliability in the present sample was good (Cronbach α = 0.773). Furthermore, to demonstrate the construct validity of our adapted mental health scale, we used the original mental health scale developed by Keyes et al.^44^ to assess participants’ stable, trait-like mental health over the last month (Cronbach α = 0.936). The final questionnaire also included measures of resilience and personality not included in the present study.

### External verification of restricted mobility

To verify that (a) the implemented lockdown measures had a local impact on the mobility of the population and (b) study participation took place during the period of restricted mobility, we harnessed the publicly-available Facebook mobility dataset from the “Data for Good” program. The dataset includes the mobility of all Facebook users that had their geo-positioning enabled and includes data for all regions where we had recruited participants. Importantly, the data are aggregated and processed with a Facebook-proprietary algorithm that ensures privacy of the people to provide an index correlating with movements of real people^48^. We used the Facebook mobility dataset and the ZIP code of participants’ current residence to assess mobility in all regions in which participants had been recruited (see Fig. 3).

### Data quality

We used *R*^49^ to examine the consistency of the demographic data assessed at the beginning of the study and after four weeks (ICC, Cohen κ, and Cramer’s ν as appropriate). In line with past research^50^, it appeared that participants had problems estimating their years spent in education as indicated by the rather low correlation between the years of education indicated at the beginning and end of the study (*r* = 0.77, *p* < 0.001). We therefore excluded the years of education variable from further analysis. We assessed the overall correlation between our adapted measure of daily mental health and somatic symptoms assessed at the end of the study as an indication of criterion validity and with trait-like mental health in the past month assessed at the end of the study as an indication of construct validity. We applied generalizability theory analysis^24,25^ to check the reliability of our daily mental health measure in our multi-level design using five random subsamples and the *multilevel.reliability* function in the *psych* R-package.

### Missing data

Visual inspection of the data indicated that participants had sometimes left the time variables (i.e., communication, physical and outside activity) blank as opposed to indicating 0 whenever they had not engaged in that behavior on that day. When a participant had responded to at least one of the time variables, we changed all time variables which had been left blank to 0 (5.8% of all questionnaires). After this procedure, the number of questionnaires with missing data was very low (1.4% of all questionnaires). Only *n* = 9 participants did not state age and/or gender.

### Log transformation of time variables

Most of the time variables were highly skewed (skewness = 2.12–13.17). We therefore log-transformed all of the time variables (1 + log), which resulted in an acceptable range ( <|1.45|) based on the recommendations of Bentler (^51^; ± 3) and Byrne (^52^; ± 5).

### Multi-level regression analysis

We used the *lme4*^53^, *lmerTest*^54^, and *sjstats*^55^ packages in *R* to calculate two-level regression models with daily assessments (level 1) nested within persons (level 2). In multi-level models, data is weighted according to participation; in our case, data from participants who submitted more daily assessments influenced the results more than the data from participants who submitted fewer daily assessments. Multi-level regression models can also handle missing data, which was in any case very low (as stated above, 1.4% of the questionnaires had missing data on level 1 and 2.2% participants had missing data for age and/or sex on level 2).

We first ran a series of separate models without any predictors and mental health and the communication variables as the outcome variables in order to calculate the respective ICC (i.e., the proportion of variance attributed to differences between participants versus day-to-day fluctuation).

Next, to analyze the association between lockdown mental health and digital communication, we calculated random-intercept, random-slope models. On level 1, we entered time spent in face-to-face, videoconferencing, digital text, and telephone communication on a particular day as the predictors, as well as daily time spent conducting physical and outside activity as control variables. On level 2, we entered each participant’s average (personal mean; pm) time spent on face-to-face, videoconferencing, digital text, and telephone communication across the four weeks as the predictors, as well as each participant’s personal mean time spent on physical and outside activity, age, age^2^ and gender as control variables. All level 1 predictors were centered around the person’s own average (centered within cluster, cwc), and age and age^2^ on level 2 were centered around the grand mean (cgm)^56–58^. We ran random-intercept random-slope models and random-intercept fixed-slope models and analyzed whether the random model had a significant better fit to the data. We successively excluded random-effects predictors one-by-one, starting with the least predictive, until there were no problems with model convergence or singular fit. The final model is displayed below and fit the data better than a pure fixed-effects model (χ^2^ = 198.5, *p* < 0.001):

Level 1 (within person):

$$\begin{aligned} {\text{Mental health}}_{{{\text{ti}}}} & = \, \pi_{{0{\text{i}}}} + \, \pi_{{{\text{1i}}}} {\text{Videoconferencing}}_{{{\text{ti}}}} .{\text{cwc }} + \, \pi_{{{\text{2i}}}} {\text{Digital text}}_{{{\text{ti}}}} .{\text{cwc }} \\ & \;\;\; + \, \pi_{{{\text{3i}}}} {\text{Face-to-face}}_{{{\text{ti}}}} .{\text{cwc }} + \, \pi_{{{\text{4i}}}} {\text{Telephone}}_{{{\text{ti}}}} .{\text{cwc }} \\ & \;\;\; + \, \pi_{{{\text{5i}}}} {\text{Physical activity}}_{{{\text{ti}}}} .{\text{cwc }} + \, \pi_{{{\text{6i}}}} {\text{Outside activity}}_{{{\text{ti}}}} .{\text{cwc }} + e_{{{\text{ti}}}} \\ \end{aligned}$$

Level 2 (between persons):

$$\begin{aligned} \pi_{{0{\text{i}}}} & = \, \beta_{00} + \, \beta_{{0{1}}} {\text{Videoconferencing}}.{\text{pm }} + \, \beta_{{0{2}}} {\text{Digital text}}.{\text{pm }} + \, \beta_{{0{3}}} {\text{Face-to-face}}.{\text{pm }} \\ & \;\;\; + \, \beta_{{0{4}}} {\text{Telephone}}.{\text{pm }} + \, \beta_{{0{5}}} {\text{Physical activity}}.{\text{pm }} + \beta_{{0{6}}} {\text{Outside activity}}.{\text{pm }} \\ & \;\;\; + \, \beta_{{0{7}}} {\text{Gender }}\left( {{\text{female}}} \right) \, + \, \beta_{{0{8}}} {\text{Age}}.{\text{cgm }} + \, \beta_{{0{9}}} \left( {{\text{Age}}.{\text{cgm}}} \right)^{{2}} + r_{{0{\text{i}}}} \\ \end{aligned}$$

$$\pi_{{{\text{1i}}}} = \beta_{{{1}0}} + r_{{{\text{1i}}}}$$

$$\pi_{{{\text{4i}}}} = \beta_{{{4}0}} + r_{{{\text{4i}}}}$$

To obtain the standardized coefficients, we used the *effect size* package^59^ which takes the different levels of standardization into account (i.e., level 1 parameters are standardized within groups, while level 2 parameters are standardized between groups)^60^. Standardized coefficients can be interpreted as effect sizes (0.1 = small, 0.3 = medium, 0.5 = large effect size). To assess the explanatory power of the model, we used *R*^2^_GLMM_^61,62^ as a measure of explained variance, which can be interpreted like the traditional *R*^2^ statistic in regression analyses. *R*^2^_conditional_ represents the proportion of variance explained by both fixed and random factors, and *R*^2^_marginal_ the proportion of variance explained by the fixed factors alone. We also calculated AIC and BIC as information criteria indices (following Nakagawa and Schielzeth^62^, as well as Ω^2^^63,64^. Ω^2^ is conceptually similar to *R*^2^ but is a less biased effect size metric that corrects the overestimation of *R*^2^ for population parameters, often resulting in somewhat smaller, more conservative estimates^65,66^.

### Multicollinearity, heteroscedasticity and non-normality of residuals

We used the *performance* package^67^ to calculate the variance inflation factor (VIF) as an indicator of multicollinearity in single-level regression models, and to check the assumptions of homoscedasticity and normality of residuals. There was no indication that multi-collinearity was a problem according to the VIF (< 1.66), but there was evidence of heteroscedasticity (*p* < 0.001) and non-normality of the residuals (*p* < 0.001). We note, however, that even minor deviations from homoscedasticity and normality are apt to be statistically significant given our large overall sample size. Graphical inspection revealed that the non-normality of residuals and heteroscedasticity was apparent only at the tails of the distribution due to floor and ceiling effects, leading to a reduced deviation of residuals.

We additionally used a perturbations approach and the *perturb* package in R^68^ to check for multi-collinearity in our multi-level data. This approach works by adding a small random perturbation value (i.e., small amounts of noise) to the respective variables and re-estimates the model by using several iterations (usually 100) to see how this affects the results. Coefficients with large standard deviations (i.e., coefficients which change a lot due to perturbations) suggest the presence of multicollinearity, although there are no clear recommendations for trigger values yet. Results are presented in Table S2.

### Mixed-effects random forest models

Due to some ambiguity as to whether the data met the assumptions of MLM (homoscedasticity, normality of residuals, no multi-collinearity), we additionally used mixed-effects random forest (MERF) models^28,29^ to double-check our results. In general, random forest (RF) models use a process called recursive partitioning^30^ to assess the importance of each predictor for a particular outcome by analyzing all possible relationships between the predictors and the outcome. This is done by drawing random subsets of predictors and participants, examining the predictive power of each predictor within the respective subset, and repeating the procedure over hundreds of bootstrap samples. To determine the overall importance of the predictor for the outcome, the predictive power of each variable across all iterations is averaged. RF models are robust to many of the problems frequently encountered with other types of analyses, such as overfitting, higher-order interactions, correlated predictors, non-linearity, or heterogeneity^69^. RF models can therefore identify the relevance of different predictors for a particular outcome with a high degree of accuracy.

To calculate the MERF models, we used the *LongituRF* package in *R*^28^ and the data from days 1 through 29. When calculating RF models, three important parameters must be specified which can impact the stability of the results^70,71^: the number of trees (*ntree*), the number of predictors randomly selected from all predictors in the model used in each tree (*mtry),* and the computational starting point for the randomization (*seed*). Consistent with standard recommendations, we set *ntree* to 1000^71–73^ and *mtry* to one third of the total numbers of predictors (in our case with 8 predictors, this would be 3;^71,74^) or the square root of the total number of predictors (in our case 3;^30,73,75^). We thus calculated five different models with five different mtry parameters (*mtry* = 3–7) in order to analyze the stability of the model. We set the seed variable to 666 but did not vary it because past research found that RFs are very robust across differing seed values^76^. We set the number of iterations to 100 iterations, and the delta (i.e., stopping rule) to 0.001^28^. Because RF models are a non-parametric procedure, we included our variables as-is into the model without centering or log-transformation.

## Supplementary Information

Supplementary Information.

## Data Availability

https://osf.io/f2epk/.
