# Supplementary material for: Face-to-face more important than digital communication for mental health during the pandemic
Source: Sci Rep. 2023 May 17;13:8022. doi: 10.1038/s41598-023-34957-4 (PMC10191089; doi:10.1038/s41598-023-34957-4)
Supplement: Supplementary file 1 — Supplementary Information. [file 41598_2023_34957_MOESM1_ESM.docx]

Supplementary Information for:

**Face-to-face more important than digital communication for mental health during the pandemic**

**This online supplement contains the following information:**

- Supplementary Methods
- Supplementary Figure S1: Number of completed questionnaires of each participant.
- Supplementary Table S1: Results of the supplementary multi-level regression analysis of face-to-face and digital communication with family, friends, and others as predictors of mental health.
- Supplementary Table S2: Impact of perturbations on the coefficients of the predictors in the multi-level regression analysis.
- Supplementary Table S3: Results of the multi-level regression analysis of face-to-face and digitally-mediated communication as predictors of mental health, but only for participants being single as their current relationship status.

Supplementary Methods

To check whether the relationships between communication and mental health depended on *with whom* people communicated, we re-ran our MLM model with three separate terms for each form of communication with family, friends, and others instead of the sum scores following the same MLM procedure as described above (see Table S1). The final model with random effects had a better fit than a pure fixed-effects model (χ^2^ = 320.1, *p* < .001). There was evidence of heteroscedasticity (*p* < .001), and non-normality of residuals (*p* < .001), but no evidence of multi-collinearity (maximum VIF was 3.53 for age). Based on the similarity of the standardized coefficients for family, friends, and others across communication types, we concluded that communication partner did not make a meaningful difference. In line with the main analysis, face-to-face communication (regardless of with whom someone communicated) was a better predictor of mental health than any form of digital communication, including videoconferencing. Furthermore, also in line with the main analysis, digital text-based communication was also a substantial predictor of mental health (between-participants as well as daily fluctuations).

**Fig. S1.**

Number of completed questionnaires of each participant.


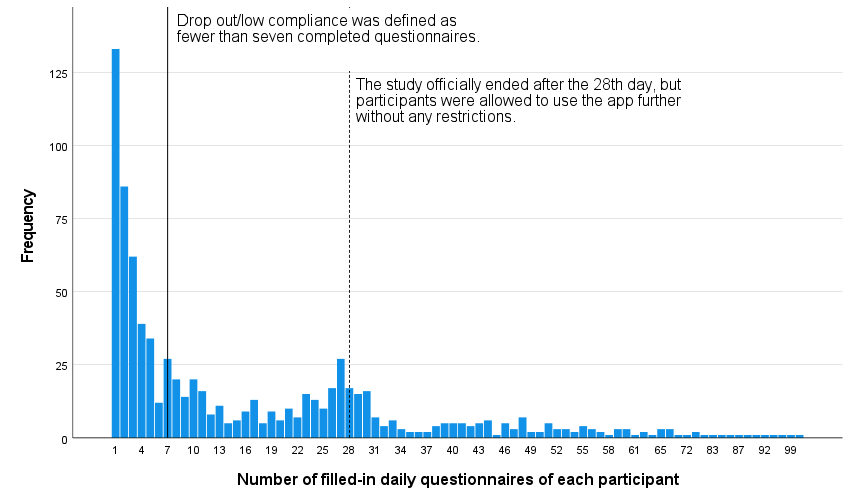


**Table S1**.

Results of the supplementary multi-level regression analysis of face-to-face and digital communication with family, friends, and others as predictors of mental health.

|  | Fixed | | | | | |  | Random | | | | |  |
| --- | --- | --- | --- | --- | --- | --- | --- | --- | --- | --- | --- | --- | --- |
|  | *B* | *CI* | β | *SE B* | | *t* |  | *SD* | | | | |  |
| Intercept | 6.09 | 4.63 – 7.55 |  | 0.74 | 8.19*** | | | | 3.28 | | |  |  |
| Within-person (all .cwc) |  |  |  |  |  | | | |  |  |  |  |  |
| Face-to-face – family^a^ | 0.15 | 0.11 – 0.19 | 0.11 | 0.02 | 7.63*** | | | | 0.20 | | |  |  |
| Face-to-face – friends^a^ | 0.15 | 0.12 – 0.18 | 0.13 | 0.02 | 9.34*** | | | | 0.18 | | |  |  |
| Face-to-face – other^a^ | 0.10 | 0.07 – 0.13 | 0.09 | 0.02 | 6.74*** | | | | 0.16 | | |  |  |
| Videoconferencing – family^a^ | 0.03 | -0.02 – 0.08 | 0.01 | 0.03 | 1.22 | | | |  |  |  |  |  |
| Videoconferencing – friends^a^ | 0.05 | 0.02 – 0.09 | 0.03 | 0.02 | 2.86** | | | |  |  |  |  |  |
| Videoconferencing – other^a^ | 0.02 | -0.01 – 0.05 | 0.01 | 0.01 | 1.32 | | | |  |  |  |  |  |
| Digital text only – family^a^ | 0.03 | -0.01 – 0.07 | 0.02 | 0.02 | 1.57 | | | |  | | |  |  |
| Digital text only – friends^a^ | 0.07 | 0.03 – 0.11 | 0.04 | 0.02 | 3.55*** | | | |  | | |  |  |
| Digital text only – other^a^ | 0.04 | 0.00 – 0.07 | 0.02 | 0.02 | 1.98* | | | |  | | |  |  |
| Telephone – family^a^ | <0.01 | -0.03 – 0.04 | <0.01 | 0.02 | 0.22 | | | |  |  |  |  |  |
| Telephone – friends^a^ | 0.01 | -0.03 – 0.05 | <0.01 | 0.02 | 0.49 | | | |  |  |  |  |  |
| Telephone – other^a^ | 0.03 | -0.01 – 0.07 | 0.02 | 0.02 | 1.48 | | | |  |  |  |  |  |
| Sport activity^a^ | 0.09 | 0.06 – 0.12 | 0.07 | 0.01 | 6.18*** | | | |  |  |  |  |  |
| Outside activity^a^ | 0.12 | 0.09 – 0.15 | 0.09 | 0.02 | 7.84*** | | | |  |  |  |  |  |
| Between-person (all .pm, except sex and age) | | | | | | | | | | | |  |  |
| Face-to-face – family^a^ | 0.23 | 0.06 – 0.39 | 0.13 | 0.09 | 2.64** | | | |  |  |  |  |  |
| Face-to-face – friends^a^ | 0.33 | 0.08 – 0.58 | 0.14 | 0.13 | 2.57* | | | |  |  |  |  |  |
| Face-to-face – other^a^ | 0.21 | -0.10 – 0.51 | 0.08 | 0.15 | 1.33 | | | |  |  |  |  |  |
| Videoconferencing – family^a^ | 0.05 | -0.44 – 0.53 | 0.01 | 0.25 | 0.19 | | | |  |  |  |  |  |
| Videoconferencing – friends^a^ | 0.08 | -0.38 – 0.53 | 0.02 | 0.23 | 0.33 | | | |  |  |  |  |  |
| Videoconferencing – other^a^ | -0.03 | -0.44 – 0.37 | -0.01 | 0.21 | -0.16 | | | |  |  |  |  |  |
| Digital text only – family^a^ | 0.18 | -0.22 – 0.58 | 0.05 | 0.21 | 0.88 | | | |  |  |  |  |  |
| Digital text only – friends^a^ | 0.34 | 0.03 – 0.66 | 0.13 | 0.16 | 2.15* | | | |  |  |  |  |  |
| Digital text only – other^a^ | 0.54 | 0.10 – 0.98 | 0.15 | 0.22 | 2.42* | | | |  |  |  |  |  |
| Telephone – family^a^ | 0.17 | -0.30 – 0.64 | 0.05 | 0.24 | 0.71 | | | |  |  |  |  |  |
| Telephone – friends^a^ | -0.18 | -0.61 – 0.24 | -0.05 | 0.22 | -0.85 | | | |  |  |  |  |  |
| Telephone – other^a^ | -0.40 | -0.97 – 0.16 | -0.09 | 0.29 | -1.40 | | | |  |  |  |  |  |
| Sport activity^a^ | 0.37 | 0.07 – 0.68 | 0.15 | 0.16 | 2.41* | | | |  |  |  |  |  |
| Outside activity^a^ | -0.26 | -0.64 – 0.13 | -0.09 | 0.20 | -1.31 | | | |  |  |  |  |  |
| Gender (female)^b^ | -0.83 | -1.70 – 0.04 | -0.09 | 0.44 | -1.87 | | | |  |  |  |  |  |
| Age.cgm | -0.02 | -0.07 – 0.02 | -0.08 | 0.02 | -1.00 | | | |  |  |  |  |  |
| (Age.cgm)^2^ | <0.01 | -0.00 – 0.00 | 0.11 | <0.01 | 1.55 | | | |  |  |  |  |  |

*Note*. *R*^2^_conditional_ = 77%, *R*^2^_marginal_ = 11%; AIC = 42438, BIC = 42746, Ω^2^ = 79% ^a^Log transformed. ^b^Reference for gender was male. **p* < .05, ***p* < .01, ****p* < .001. cwc = centered within clusters. pm = personal mean. cgm = centered around the grand mean.

**Table S2**.

Impact of perturbations on the coefficients of the predictors in the multi-level regression analysis.

|  | *M* | *SD* | min | max |
| --- | --- | --- | --- | --- |
| Fixed |  |  |  |  |
| Intercept | 8.196 (8.504) | 0.574 (0.430) | 4.438 (4.438) | 8.708 (9.094) |
| Face-to-face.cwc | 0.151 (0.075) | 0.022 (0.022) | 0.126 (0.048) | 0.282 (0.282) |
| Videoconferencing.cwc | 0.036 (0.018) | 0.007 (0.008) | 0.025 (-0.005) | 0.056 (0.056) |
| Digital text.cwc | 0.018 (0.011) | 0.012 (0.009) | -0.013 (-0.009) | 0.040 (0.040) |
| Telephone.cwc | 0.014 (0.007) | 0.006 (0.007) | 0.000 (-0.012) | 0.032 (0.027) |
| Physical activity.cwc | 0.066 (0.048) | 0.008 (0.008) | 0.046 (0.024) | 0.081 (0.073) |
| Outside activity.cwc | 0.104 (0.068) | 0.009 (0.009) | 0.087 (0.051) | 0.127 (0.126) |
| Face-to-face.pm | 0.024 (0.008) | 0.072 (0.050) | -0.032 (-0.036) | 0.494 (0.494) |
| Videoconferencing.pm | 0.011 (0.008) | 0.049 (0.034) | -0.033 (-0.021) | 0.323 (0.323) |
| Digital text.pm | 0.020 (0.009) | 0.069 (0.049) | -0.048 (-0.026) | 0.480 (0.480) |
| Telephone.pm | 0.003 (0.000) | 0.029 (0.014) | -0.046 (-0.028) | 0.073 (0.073) |
| Physical activity.pm | 0.013 (0.005) | 0.039 (0.027) | -0.044 (-0.018) | 0.247 (0.247) |
| Outside activity.pm | 0.008 (0.000) | 0.022 (0.011) | -0.040 (-0.022) | 0.058 (0.042) |
| Gender (female) | -0.088 (-0.076) | **0.213 (0.164)** | -1.306 (-1.306) | 0.174 (0.223) |
| Age.cgm | -0.037 (-0.015) | 0.012 (0.010) | -0.059 (-0.050) | -0.009 (0.010) |
| (Age.cgm)^2^ | 0.003 (0.002) | 0.000 (0.000) | 0.001 (0.001) | 0.003 (0.003) |
| Random |  |  |  |  |
| Intercept | 3.471 (3.499) | 0.031 (0.025) | 3.293 (3.293) | 3.506 (3.532) |
| Face-to-face.cwc | 0.195 (0.108) | 0.020 (0.025) | 0.167 (0.066) | 0.307 (0.307) |
| Digital text.cwc | 0.121 (0.062) | 0.039 (0.030) | 0.034 (0.006) | 0.212 (0.212) |
| Intercept × Digital text.cwc | -0.447 (-0.478) | **0.233 (0.295)** | -1.000 (-1.000) | -0.221 (-0.013) |
| Intercept × Face-to-face.cwc | -0.244 (-0.244) | 0.039 (0.067) | -0.316 (-0.480) | -0.135 (-0.074) |
| Digital text.cwc × Face-to-face.cwc | 0.186 (0.267) | **0.164 (0.361)** | -0.099 (-0.854) | 0.562 (1.000) |
| Residual | 1.992 (2.023) | 0.007 (0.009) | 1.960 (1.960) | 2.002 (2.043) |

*Note*. All time-based variables (communication, physical and outside activity) were log transformed. For adding noise to the data, a standard deviation of 1 was used for metric variables and 95% probability of re-classifying into the same category for gender (50 iterations). For comparison, in parentheses are the values for added noise with *SD* = 2 and 100 iterations. Standard deviations with effects larger than 0.1 are in bold. cwc = centered within clusters. pm = personal mean. cgm = centered grand mean.

**Table S3.**

Results of the multi-level regression analysis of face-to-face and digitally-mediated communication as predictors of mental health, but only for participants being single as their current relationship status.

|  | | Fixed | | | | | | | | | | |  | Random | | |
| --- | --- | --- | --- | --- | --- | --- | --- | --- | --- | --- | --- | --- | --- | --- | --- | --- |
|  |  | Coeff. | | *B* | | *CI B* | β | | *SE B* | *t* | | |  | Coeff. | *SD* | |
| Intercept | | β_00_ | | 4.08 | | 1.73 – 6.43 | .00 | | 1.20 | 3.41*** | | |  | *r*_0_*_i_* | 3.14 | |
| Within-person (all .cwc) | | | | | | | | | | | | | | | | |
| Face-to-face^a^ | β_10_ | | 0.32 | | 0.24 – 0.40 | | .23 | | 0.04 | 8.12*** | | |  | *r*_1_*_i_* | 0.25 | |
| Videoconferencing^a^ | | β_20_ | | 0.06 | | 0.02 – 0.09 | .05 | | 0.02 | 2.94** | | |  |  |  |  |
| Digital text^a^ | | β_30_ | | 0.10 | | 0.02 – 0.18 | .05 | | 0.04 | 2.38* | | |  | *r*_3_*_i_* | 0.31 |  |
| Telephone^a^ | | β_40_ | | 0.08 | | 0.03 – 0.13 | .05 | | 0.02 | 3.41*** | | |  |  |  |  |
| Sport activity^a^ | | β_50_ | | 0.09 | | 0.04 – 0.14 | .07 | | 0.02 | 3.73*** | | |  |  |  |  |
| Outdoor activity^a^ | | β_60_ | | 0.10 | | 0.05 – 0.15 | .07 | | 0.03 | 3.85*** | | |  |  |  |  |
| Between-person (all .pm, except gender and age) | | | | | | | | | | | | | | | | |
| Face-to-face^a^ | β_07_ | | 0.54 | | 0.13 – 0.95 | | | .22 | 0.21 | | 2.59* | |  |  |  | |
| Videoconferencing^a^ | | β_01_ | | 0.18 | | -0.24 – 0.59 | | .07 | 0.21 | | | 0.84 |  |  |  | |
| Digital text^a^ | | β_04_ | | 0.78 | | 0.30 – 1.27 | | .25 | 0.25 | | | 3.15** |  |  |  | |
| Telephone^a^ | | β_010_ | | 0.43 | | -0.07 – 0.93 | | .14 | 0.26 | | | 1.68 |  |  |  | |
| Sport activity^a^ | | β_013_ | | 0.42 | | -0.03 – 0.88 | | .17 | 0.23 | | | 1.84 |  |  |  | |
| Outdoor activity^a^ | | β_014_ | | -0.64 | | -1.21 – -0.06 | | -.24 | 0.29 | | | -2.17* |  |  |  | |
| Gender (female)^b^ | | β_015_ | | -1.62 | | -2.82 – -0.42 | | -.19 | 0.61 | | | -2.64** |  |  |  | |
| Age.cgm | | β_016_ | | -0.01 | | -0.08 – 0.06 | | -.12 | 0.03 | | | -0.29 |  |  |  | |
| (Age.cgm)^2^ | | β_017_ | | 0.01 | | 0.00 – 0.01 | | .29 | 0.01 | | | 2.44* |  |  |  | |

*Note*. *R*^2^_conditional_ = 76%, *R*^2^_marginal_ = 19%; AIC = 17152, BIC = 17296, Ω^2^ = 77% ^a^Log transformed. ^b^Reference group for gender was male. cwc = centered within cluster. pm = personal mean. cgm = centered around grand mean. **p* < .05, ***p* < .01, ****p* < .001.
